# Phospholipase A_2_ – nexus of aging, oxidative stress, neuronal excitability, and functional decline of the aging nervous system? Insights from a snail model system of neuronal aging and age-associated memory impairment

**DOI:** 10.3389/fgene.2014.00419

**Published:** 2014-12-04

**Authors:** Petra M. Hermann, Shawn N. Watson, Willem C. Wildering

**Affiliations:** ^1^Department of Biological Sciences, University of CalgaryCalgary, AB, Canada; ^2^Department of Physiology and Pharmacology, University of CalgaryCalgary, AB, Canada; ^3^Hotchkiss Brain Institute, University of CalgaryCalgary, AB, Canada

**Keywords:** aging, age-associated memory impairment (AMI), lipid peroxidation, phospholipases A2, neuronal excitability, neuronal plasticity, *Lymnaea stagnalis*

## Abstract

The aging brain undergoes a range of changes varying from subtle structural and physiological changes causing only minor functional decline under healthy normal aging conditions, to severe cognitive or neurological impairment associated with extensive loss of neurons and circuits due to age-associated neurodegenerative disease conditions. Understanding how biological aging processes affect the brain and how they contribute to the onset and progress of age-associated neurodegenerative diseases is a core research goal in contemporary neuroscience. This review focuses on the idea that changes in intrinsic neuronal electrical excitability associated with (per)oxidation of membrane lipids and activation of phospholipase A_2_ (PLA_2_) enzymes are an important mechanism of learning and memory failure under normal aging conditions. Specifically, in the context of this special issue on the biology of cognitive aging we portray the opportunities offered by the identifiable neurons and behaviorally characterized neural circuits of the freshwater snail *Lymnaea stagnalis* in neuronal aging research and recapitulate recent insights indicating a key role of lipid peroxidation-induced PLA_2_ as instruments of aging, oxidative stress and inflammation in age-associated neuronal and memory impairment in this model system. The findings are discussed in view of accumulating evidence suggesting involvement of analogous mechanisms in the etiology of age-associated dysfunction and disease of the human and mammalian brain.

## INTRODUCTION

Most organisms age, humans and the vast majority of other complex animals included. That is, the life functions required for their survival and reproduction weaken as they grow older due to natural processes that are still only partially understood and involve a complex combination of chemical, genetic, metabolic, molecular, physiological, ecological, and evolutionary features (see for instance Kirkwood and Austad, 2000; Ljubuncic and Reznick, 2009; Masoro and Austad, 2011).

Aging generally affects all aspects of an organism’s biology including, in the case of humans and complex multicellular animals, the nervous system’s ability to execute its control processes and support learning and memory functions. For example, declining learning and memory performance, weakening sensory, and motor functions, and slowing of reaction times are common symptoms of old age in humans and many vertebrate and invertebrate model systems (Burke and Barnes, 2006; Chawla and Barnes, 2007; Murakami, 2007; Partridge, 2008; Yeoman et al., 2012; Haddadi et al., 2014). Some of these changes, particularly in our own species, may involve pathological processes such as Alzheimer’s and Parkinson’s disease. However, even in the healthy aging brain where loss of neurons and circuits to cell death is generally not a significant factor, functional performance tends to decline with age (Peters et al., 1994; West et al., 1994; Rapp and Gallagher, 1996; Rasmussen et al., 1996; Gazzaley et al., 1997; Merrill et al., 2000, 2001; Keuker et al., 2003; Burke and Barnes, 2006). Why this happens, particularly what makes healthy aging neurons change the way in which they work and communicate with each other is the focus of this article. Specifically, we explore the idea that processes triggered by oxidative stress ensuing at the level of phospholipid bilayer membranes of cells and organelles play a central role in age-associated deterioration of the healthy aging nervous system (Note: while this review centers on non-pathological mechanisms of brain aging it should be kept in mind that many consider normal and pathological aging as two causally related extremes of a continuum in which it is not always clear where normal aging ends and pathological aging starts. See for instance Hung et al., 2010; Dai et al., 2014).

**FIGURE 1:**
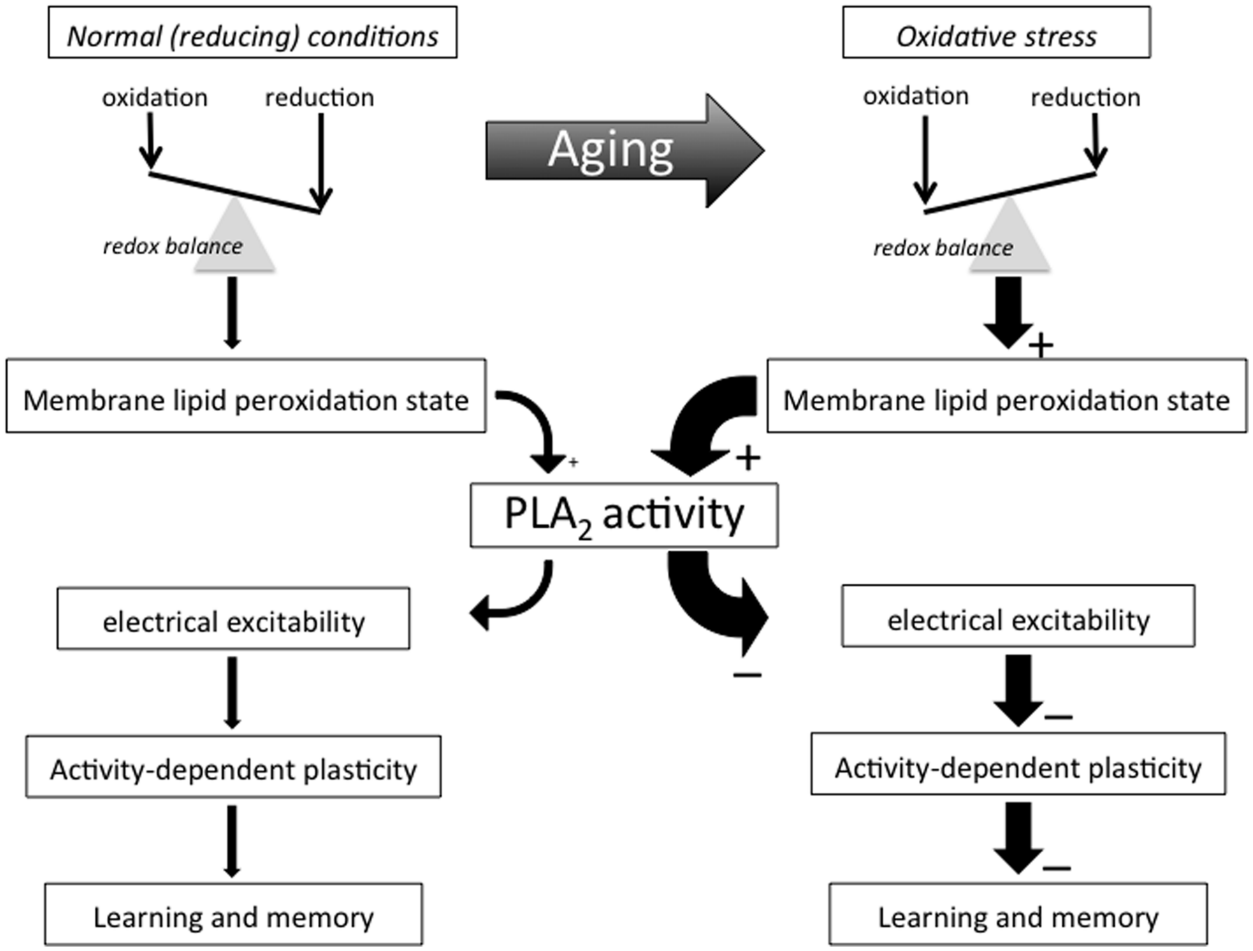
**Conceptual framework.** This flow diagram outlines key concepts and hypotheses addressed in this essay and illustrate the way we propose they contribute to AMI. Many important biological processes involve redox reactions and many of life’s macromolecules are sensitive to electrophilic attacks that may impair their ability to perform their normal functional or structural roles. Few of life’s building blocks are as sensitive to oxidation as the poly-unsaturated fatty acids (PUFAs) making up much of cell and organelle membranes. Organisms generate electrophiles like reactive oxygen and nitrogen species as part of many essential live processes, including metabolism, host defense, and intercellular signaling processes. Active maintenance of an appropriate redox balance is therefore of the utmost importance for organismal function and survival. Biological aging tends to be associated with shifts in redox balance away from reducing towards oxidative conditions in many of an organism’s molecular, cellular, and organismal systems and domains, including an advanced state of membrane PUFA peroxidation that recruits PLA_2_ and induces a (PLA_2_-dependent) decline in neuronal excitability and activity-dependent plasticity that manifests itself as AMI.

**FIGURE 2:**
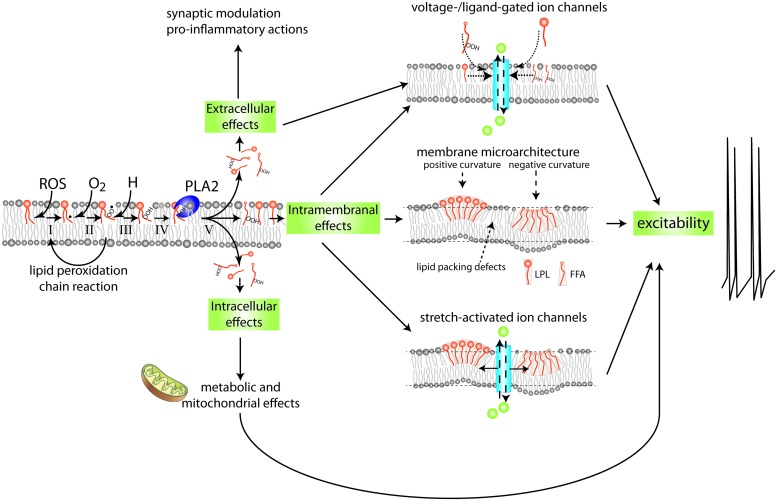
**Mechanism and possible implications of membrane lipid peroxidation and phospholipase A_2_ activation.** PUFAs are highly susceptible to (per)oxidation. Peroxidized PUFAs are prone to excision by members of the PLA_2_ family of enzymes. The products of PLA_2_’s enzymatic activity, free fatty acids (FFAs) and lysophospholipids (LPLs), have a wide range of extracellular, intracellular, and intramembranal biological activities. This highly simplified diagram indicates a few of the mechanisms and targets with special relevance to the nervous system. FFAs and LPLs can themselves act as signaling molecules and modify gating behavior of various types of ion channels. In addition they can act as substrate for other lipid-based intra- and extracellular signaling cascades, including signaling systems regulating inflammation. Moreover, through their effect on membrane microarchitecture they may change functions of integral membrane proteins such as stretch-activated ion channels, alter lipid micro domain organization, disrupt organization of transmembrane signaling complexes, or alter other cellular processes such as membrane fission and fusion that are important in the regulation of intrinsic neuronal excitability, synaptic transmission, and molecular mechanisms underlying neuronal plasticity. See **Figure 6** for details on mechanisms involved in PUFA peroxidation.

The idea that oxidative stress is a major factor in biological aging has a long history and extensive experimental support (Harman, 1956; Pérez et al., 2009; Hekimi et al., 2011; Speakman and Selman, 2011; Zimniak, 2011; Alcedo et al., 2013). Yet, despite its prominence in the conceptual framework of biological aging, understanding of how oxidative stress translates to functional decline of the aging nervous system remains incomplete. Here we examine how (per)oxidation of methylene-interrupted poly-unsaturated fatty acids^1^ (PUFAs) and subsequent activation of members of the phospholipase A_2_ (PLA_2_) family of fatty acylases may play a central role in declining excitability of aging neurons and age-associated memory impairment (AMI; see **Figure 1** for conceptual framework; see **Figure 2** for mechanisms and potential implications of PUFA peroxidation).

## NEUROBIOLOGICAL CORRELATES OF NORMAL AGING

Considerable effort has been put into identifying neurobiological correlates of normal brain aging for several decades. The picture emerging from these studies is that structural and functional changes occurring in the normal aging brain are generally much more low-key and variable than those found in pathological aging brains. The list of physiological and biochemical symptoms of neuronal aging is extensive and includes evidence for oxidative stress, impaired energy and redox metabolism, Ca^2+^ signaling perturbation, accumulating damage to proteins, lipids, nucleic acids and organelles, declining dendritic complexity, alterations in synaptic density, distribution and function, changes in gene regulation, and molecular processes underlying remodeling of synapses (Barnes, 1994; Burke and Barnes, 2006; Mattson and Magnus, 2006; Esiri, 2007; Shankar, 2010; Toescu and Vreugdenhil, 2010; Yeoman et al., 2012).

While cell autonomous processes likely constitute an important facet of neuronal aging, evidence for significant cell non-autonomous (i.e., organismal) dimensions of neuronal aging is growing. For instance, it is evident that brain aging has vascular, immunological and endocrinological dimensions (Joseph et al., 2005; Aïd and Bosetti, 2011; Yirmiya and Goshen, 2011; Alcedo et al., 2013; Rincon and Wright, 2013; Sadagurski and White, 2013; Oakley and Tharakan, 2014; Tarumi and Zhang, 2014). One particularly intriguing organismal dimension to normal brain aging emerging in recent decades is the concept that the brain at least in part regulates its own destiny through insulin-like peptide signaling systems coupling nutrient availability and dietary conditions to the control of cell and organismal metabolism (e.g., Alcedo et al., 2013; Sadagurski and White, 2013). Furthermore, while there seems little doubt that many of the phenomena associated with neuronal aging are of a deteriorative nature, evidence for functional preservation and compensation has also been reported (see Barnes, 1994; Burke and Barnes, 2006 for review).

## THE NEED FOR SIMPLIFICATION: *Lymnaea stagnalis* AS A MODEL FOR INTEGRATIVE MOLECULE-TO-BEHAVIOR INVESTIGATIONS OF NEURONAL AND BEHAVIORAL AGING

**FIGURE 3:**
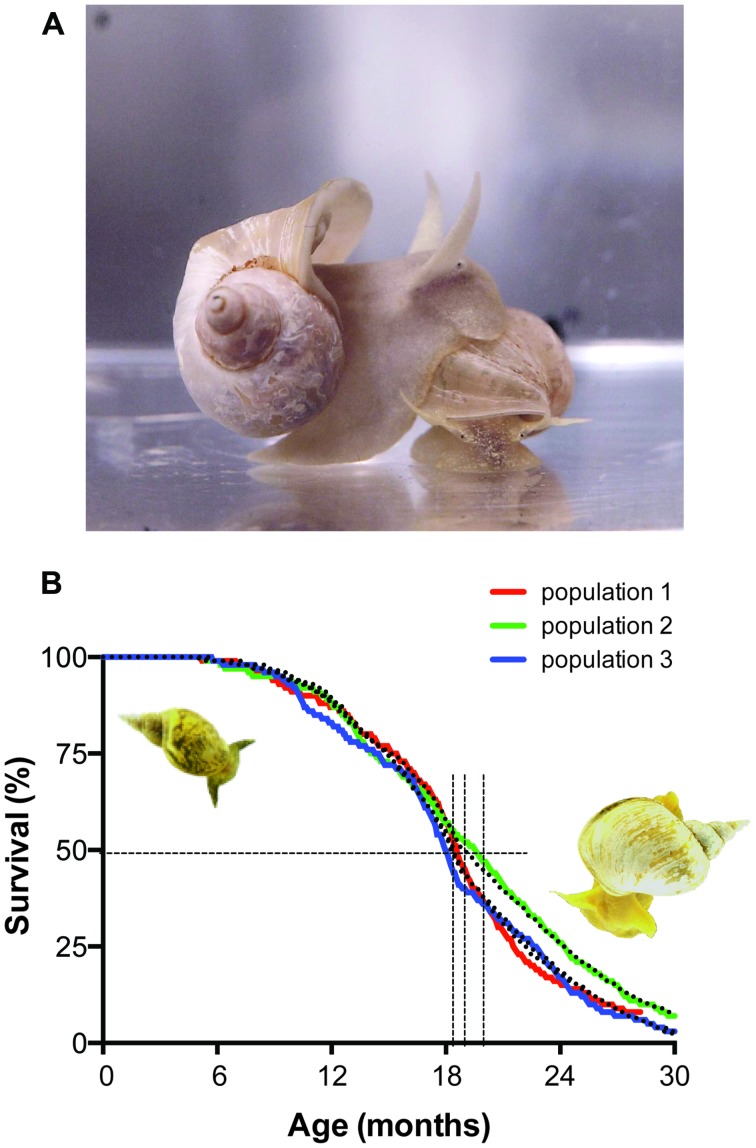
***Lymnaea stagnalis*, the animal and its survival characteristics. (A)** *L. stagnalis* is an obligate freshwater aquatic gastropod of the clade *Euthyneura*. The animals are bimodal breathers capable of absorbing oxygen through their skin and through ventilating a simple lung cavity to air. **(B)** Survival characteristics of populations of *Lymnaea* held in our aquatic facility. Shown are the census data for three different populations, representative for normal population survival in the facility (colored lines) fitted by a Weibull power law failure model (dotted lines). Under the conditions in our facility the median population age hovers around 1.5 years (vertical dotted lines median age ± 95% confidence interval) and individual maximum age routinely exceeds 2 years. Under standard culture conditions we maintain in our in house facility (i.e., *ad libitum* feeding of standard diet at 17–19°C water temperature under a 12:12 light:dark regimen) survival characteristics of healthy captive populations show very little variation and reach an average median age of 593 ± 13 days and average maximal age of 880 ± 10 days (arithmetic means ± SE of the mean, *n* = 15; for more details on aquaculture conditions see Watson et al., 2012a).

How all dietary, (bio)chemical, metabolic, molecular, cellular, physiological, system-, and organismal-level processes factor into biological aging of neurons and the nervous system and contribute to AMI remains one of the most important unsolved puzzles of neuroscience. Answering fundamental questions of this kind in a system as complex, dynamic, and inherently adaptive as the nervous system is not a trivial problem. With the goal of reducing the complexity of such undertaking, we adopted the pond snail *L. stagnalis* as our research platform (**Figure 3**). Since *Lymnaea* is not as widely known as other model systems in neuronal aging and AMI we will briefly summarize its most relevant features before continuing with the main focus of this review.

**FIGURE 4:**
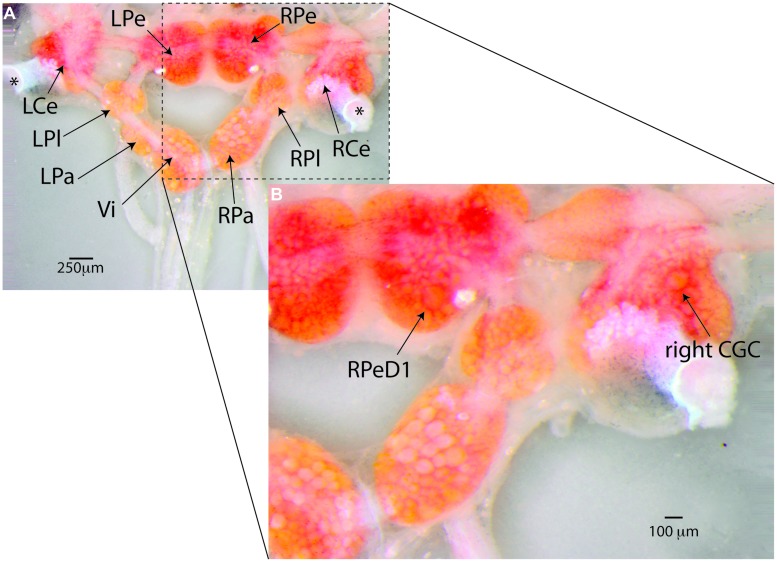
***Lymnaea’s* central nervous system and its identified neurons. (A)** *Lymnaea’s* central nervous system (CNS) is comprised of a circumesophagal ganglionic ring made up of nine ganglia plus two buccal ganglia that are not part of the inner ganglionic ring (LCe, left cerebral ganglion; RCe, right cerebral ganglion; LPe, left pedal ganglion; RPe, right pedal ganglion; LPl, left pleural ganglion; RPl, right pleural ganglion; LPa, left parietal ganglion; RPa, right parietal ganglion; Vi, visceral ganglion; the paired left and right buccal ganglia are not shown in this image. To expose location of neurons discussed in this paper, the cerebral commissure connecting LCe and RCe has been cut and LCe and RCe have been folded out laterally). Inside the ganglia somata of many neurons can be recognized. Many of these neurons are individually identifiable. **(B)** Enlargement of the boxed area in **(A)** showing the location of the two identified neurons that are the main focus of the paper. Right pedal dorsal 1 (RPeD1), located on dorsal surface of the RPe ganglion is a multi-modal neuron involved in the control of *Lymnaea’s* aerial respiration and an important locus of operant-conditioning induced plasticity of the animals’ respiratory behavior. The cerebral giant cell (GCG) indicated here is one of a bilaterally symmetrical pair of interneurons instrumental in the control of *Lymnaea’s* feeding behavior. The CGCs are important loci of reward-conditioning of the animals feeding behavior. These composite images were generated from 15–20 images taken at different focal depths using DMap focus stacking algorithm in Zerene Stacker build T201404082055 (Zerene Systems, Richland, WA, USA).

*Lymnaea stagnalis* is a freshwater pulmonate snail with a long and varied track record as a model system in a wide range of basic and applied biological research, including the study of behavioral, neural, and molecular aspects of associative learning and memory. It has a relatively simple central nervous system (CNS, **Figure 4**) of about 20,000–25,000 neurons organized in 11 ganglia. The CNS contains many individually identifiable neurons and well-mapped, behaviorally characterized neural circuits supporting direct behavior-to-molecule inferences about the foundations of neuronal function and dysfunction (see for instance Benjamin et al., 2000; Taylor and Lukowiak, 2000; Brembs, 2003; Benjamin and Kemenes, 2010) including the biochemical, molecular, cell biological, and physiological foundations of neuronal aging and AMI.

Under laboratory conditions, *Lymnaea* can be effectively reared to ages and quantities required for aging research. Depending on variables like feeding regimen, animal density, day length and water temperature, the maximal age of the animals routinely exceeds 2.5 years with median ages of around 1.5 years under these conditions and age-dependent mortality follows Gompertz and Weibull survival characteristics indicative of aging (**Figure 3B**; see also Janse et al., 1988; Slob and Janse, 1988).

*Lymnaea*’s application in aging studies dates back to the 1980s with the publication of several neurophysiological studies (Frolkis et al., 1984; Nagy et al., 1985; Janse et al., 1986), the first report of age effects on associative memory performance (Audesirk et al., 1982) and work on the general biology and population-level aspects of the species’ aging process (Janse et al., 1988; Slob and Janse, 1988). Since then publications on various cell biological, endocrinological, neurophysiological, biophysical, behavioral, and reproductive aspects of aging in *Lymnaea* have appeared (Frolkis et al., 1989, 1991; Janse et al., 1989, 1999; Wildering et al., 1991; Klaassen et al., 1998; Janse and van der Roest, 2001; Arundell et al., 2006; Patel et al., 2006, 2010). Its use in research of the molecular and cellular foundations of AMI started in earnest with Hermann et al. (2007) followed by a series of other publications from our laboratory linking AMI and neuronal excitability changes to oxidative stress, inflammation, lipid peroxidation and declining glutathione (GSH) availability, and identifying PLA_2_ as a central player in these phenomena (Watson et al., 2012a,b, 2013, 2014; Hermann et al., 2013; Beaulieu et al., 2014).

**FIGURE 5:**
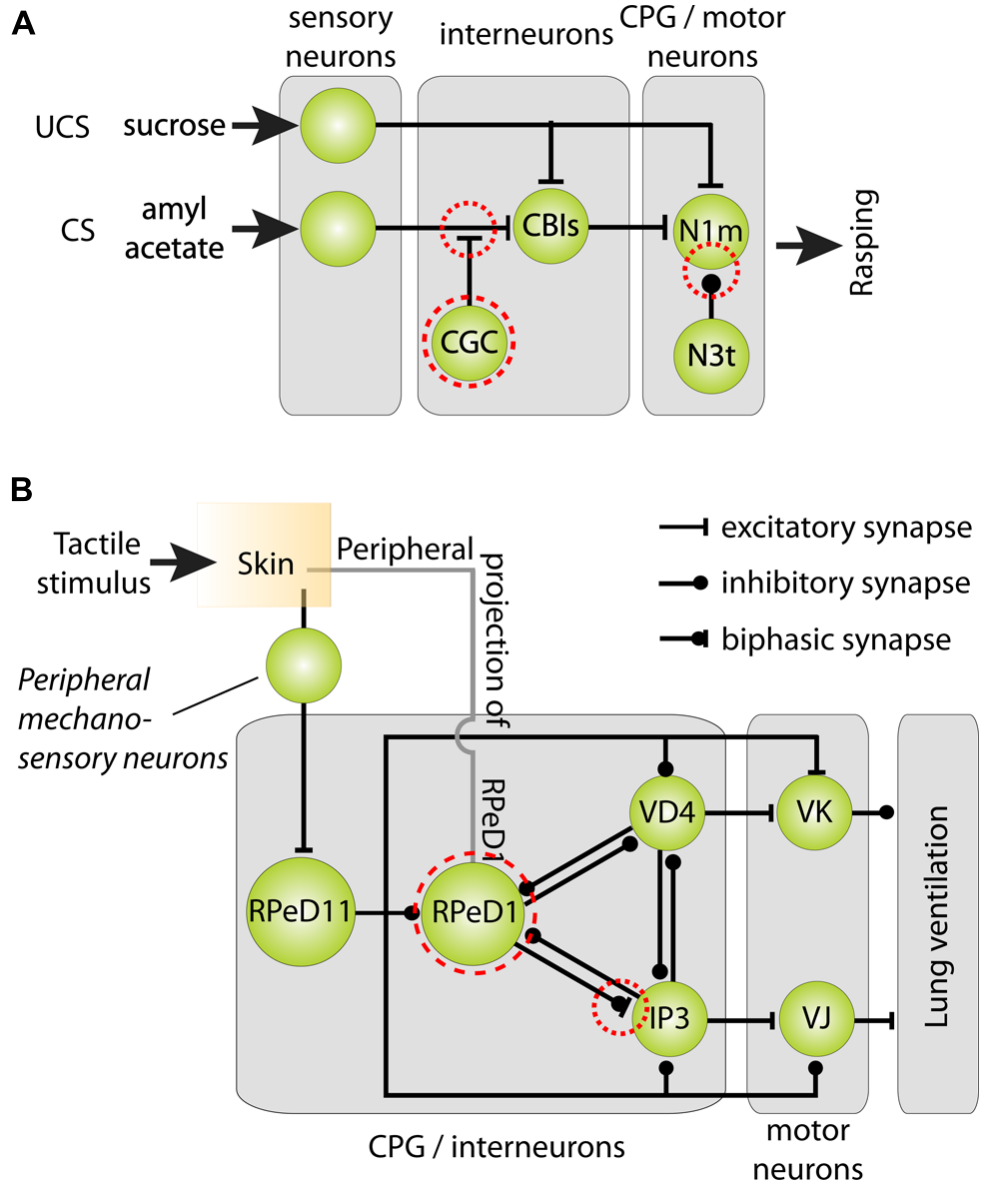
**Cellular models of classical reward and operant aversive conditioning in *Lymnaea*. (A)** Parsimonious model of the neural mechanisms underlying classical chemical reward conditioning of *Lymnaea’s* feeding behavior. Behavioral conditioning by peripheral application of sucrose as unconditioned stimulus (UCS) and amylacetate as conditioned stimulus (CS) induces a delayed persistent depolarization of the cerebral giant cells (CGC). This depolarization facilitates throughput of peripheral CS connections with cerebral-buccal interneurons (CBIs) thereby gating throughput of the CS to Neuron 1 medial (N1m) interneurons of the feeding circuit’s central pattern generator (CPG) that play a pivotal role in the initiation of the three phase rasping cycle. At the same time, the spike frequency of the CPG interneuron N3t is reduced after behaviorally conditioning thereby releasing N1m from its inhibition. **(B)** Parsimonious model of the neural mechanisms underlying aversive operant conditioning of *Lymnaea’s* aerial respiratory behavior. Aerial respiration involves opening and closing of an orifice giving access to the lung cavity (the pneumostome). The CPG driving the animals’ aerial respiration behavior consists of three identified interneurons: right pedal dorsal 1 (RPeD1), visceral dorsal 4 (VD4) and (wide-acting) input 3 neuron (IP3). Spiking activity in RPeD1 initiates rhythmic pattern of spiking activity in VD4 and IP3 that in turn trigger electrical activity in, respectively, Visceral K (VK) pneumostome closer motor neurons and visceral J (VJ) pneumostome opener motor neurons. Aversive operant conditioning through tactile stimulation of the pneumostome area induces a hyperpolarization of RPeD1 and reduces the latter’s ability to trigger rhythmic activity in IP3. Mechanosensory conditioning of the respiratory CPG’s electrical activity through tactile stimulation of the pneumostome area involves both afferent projections of RPeD1 (“peripheral projections of RPeD1”) and excitation of the ‘whole-body withdrawal interneuron’ RPeD11 by identified mechanosensory neurons. Dashed red circles in both circuit diagrams indicate neural loci of plasticity. Circuit diagrams shown were adapted from Benjamin et al. (2000), Taylor and Lukowiak (2000) and Benjamin and Kemenes (2010).

*Lymnaea* has quite a broad behavioral and sensory repertoire and can be taught to change many of its behaviors in response to a variety of chemical, mechanical, and even visual cues (Audesirk et al., 1982; Lukowiak et al., 1996; Sakakibara et al., 1998; Kawai et al., 2004; reviewed in Benjamin et al., 2000; Brembs, 2003; Lukowiak et al., 2006; Benjamin, 2012). Neurobiological substrates of several behavioral paradigms of associative learning and memory have been well characterized. In recent decades most research has focused on two training paradigms: classical conditioning of the animals’ feeding behavior, a paradigm with critical involvement of a pair of interneurons called cerebral giant cells (CGCs), and operant conditioning of their aerial respiration, a paradigm with a multi-modal neuron called right pedal dorsal 1 (RPeD1) at its center (**Figures 4** and **5**; Spencer et al., 1999; Staras et al., 1999; for detailed review on the mechanisms and circuitry underlying these behaviors and learning paradigms see Benjamin et al., 2000; Brembs, 2003; Lukowiak et al., 2006; Benjamin, 2012). Using these two learning models, detailed insights in the molecular, cellular, neuronal network, and behavioral aspects of learning and memory have been obtained (Ribeiro et al., 2003, 2005; Kemenes et al., 2006a; Nikitin et al., 2006; Sadamoto et al., 2010; Wan et al., 2010; Dalesman and Lukowiak, 2012; Rosenegger and Lukowiak, 2013; reviewed in Benjamin et al., 2000 and Benjamin, 2012). Importantly, these studies show that, like other invertebrates, *Lymnaea* learning shares many key behavioral, molecular, and functional facets with learning in vertebrate model systems.

## LIPID MEMBRANES AS A KEY THEATER OF AGING AND AGE-ASSOCIATED DECLINE OF THE NERVOUS SYSTEM

A large body of evidence indicates that oxidative stress is a prominent feature of both pathological and non-pathological forms of nervous system aging (Choi, 1995; Mattson and Magnus, 2006; Torres et al., 2011; Lopez et al., 2013; Orr et al., 2013; Haddadi et al., 2014). How oxidative stress contributes to age-associated disease processes of the brain and translates to functional decline of normal healthy aging neurons is not well understood. Here we explore the theory that processes associated with the high level of unsaturation of the lipid portion of neuronal membrane constitute a key link between aging, oxidative stress, and the age-associated physiological and behavioral decline frequently observed in normal healthy aging animals and the etiology of age-associated disorders of the brain. Central to this idea are the inordinate sensitivity of methylene-interrupted PUFAs to electrophilic attacks and subsequent activation of PLA_2_’s, a family of enzymes involved in maintenance of membrane phospholipid integrity and membrane-associated lipid signaling processes (**Figure 2**).

Eukaryote cell and organelle membranes are functionally complex, dynamically regulated assemblies of mostly glycerophospholipids, fatty acids (FAs), sterols, proteins, glycoproteins, and glycolipids (Alberts et al., 2002). While most of life’s structures are sensitive to oxidation, few are as susceptible to oxidative damage as phospholipid bilayer membranes (Zimniak, 2011). This elevated susceptibility to oxidation is due to a number of factors that conspire, as a manner of speaking, to create “perfect storm” conditions. First, under normal conditions solubility of molecular oxygen and many reactive oxygen and nitrogen species (RONS) in the lipid microenvironment of membranes is quite high (Subczynski and Wisniewska, 2000). Second, as mentioned, PUFAs are inordinately sensitive to attack by RONS and other electrophiles (Hulbert et al., 2007). Third, PUFAs that have undergone free radical attack are themselves reactive free radicals and are, because of the tightly packed molecular arrangement of phospholipids in bilayer membranes, capable of propagating potentially catastrophic lipid peroxidation cascades (Forlenza et al., 2007; Ballatori et al., 2009; Currais and Maher, 2013; Sultana et al., 2013). Neuronal membranes contain high levels of PUFAs such as arachidonic acids (AA), docosahexaenoic acids (DHA), and eicosapentaenoic acids (EPA), and are therefore particularly sensitive to (per)oxidation making lipid peroxidation potentially one of the major instruments of free radical-mediated injury and cell death in the nervous system.

Parenthetically, the idea that phospholipid membranes are one of the primary molecular theaters of aging and longevity has gained substantial traction in recent decades (for reviews see Pamplona, 2008; Zimniak, 2011). In fact, the observation that the level of lipid membrane unsaturation correlates inversely with longevity in mammals, birds, and even invertebrates led to formulation of the “Membrane hypothesis of aging” which essentially sees lipid membranes as one of the weakest and therefore highest maintenance links in the biochemical armature of living organisms (Hulbert et al., 2007; Ungvari et al., 2011, 2013; Munro and Blier, 2012; Munro et al., 2013).

## LIPID PEROXIDATION INDUCED PHOSPHOLIPASE A_2_ ACTIVITY AND ITS CONSEQUENCES

One characteristic cellular response triggered by lipid peroxidation is activation of one or more PLA_2_ family members that catalyze cleavage of FAs from the sn-2 position of glycero-phospholipids in plasma- and organelle membranes (Brown et al., 2003; Sun et al., 2004; Farooqui and Horrocks, 2006; Chen et al., 2008; Dennis et al., 2011). Although the precise underlying molecular mechanisms of lipid-peroxidation induced PLA_2_ activation are still a matter of debate (see for instance Greenberg et al., 2008), it is generally thought to involve peroxidation induced changes in fatty acyl chain geometry (Niki, 1990; Salgo et al., 1992; Rashba-Step et al., 1997; Greenberg et al., 2008).

**FIGURE 6:**
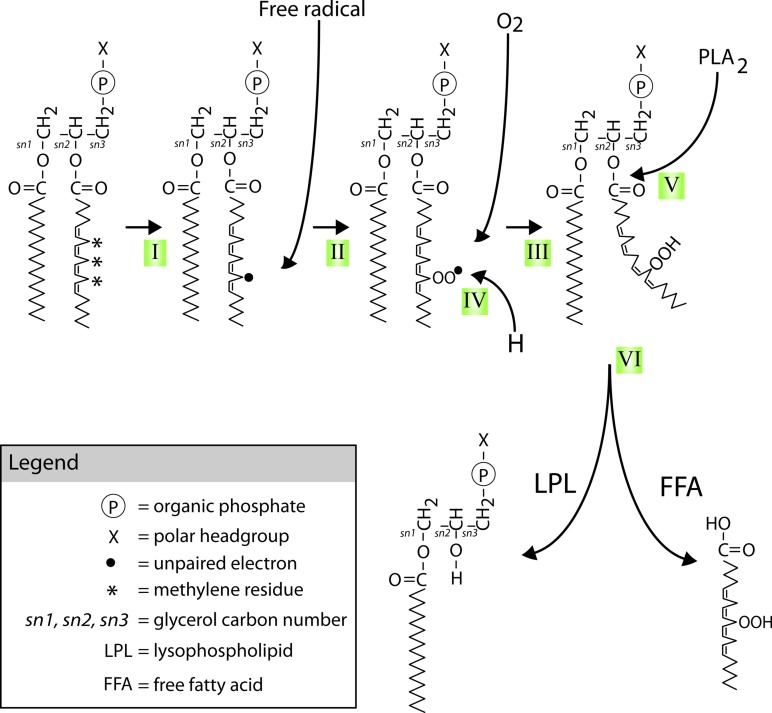
**Mechanisms of PUFAs peroxidation and hydrolysis.** Neuronal glycerophospholipids contain high levels of methylene-separated PUFAs such as the 20:4 omega-6 fatty acid arachidonic acid (AA) shown attached at the second carbon of the phospholipid glycerol backbone (the sn-2 position) in the diagram. These PUFAs are very sensitive to free radical attack at their bis-allylic C-H bonds (indicated by *) and subsequent (per)oxidation. This process starts with (I) the formation of a C-centered radical (indicated by •) followed by (II) recombination with molecular oxygen that is normally present in abundance in lipid bilayers. The lipid peroxide radicals formed in step II can start a peroxidation chain reaction unless (IV) defused by reduction through for instance donation of a proton by vitamin E or neutralization by other chemical mechanisms. Peroxidation of these PUFAs (V) recruits members of the PLA_2_ family of phospholipases. PLA_2_ mediated hydrolysis of peroxidized PUFAs (VI) generates peroxidized FFAs and LPLs.

Activation of PLA_2_ leads to the production of (peroxidized) free FAs (FFAs) and lysophospholipids (LPLs), both classes of molecules with potentially substantial biological activity (**Figure 6**; see below; Brown et al., 2003; Farooqui and Horrocks, 2006). For example, the molecular geometry of LPLs and FFAs is quite different from their mutual parent molecule (i.e., a phospholipid). They may therefore also cause changes in the micro-architecture and biophysical properties of phospholipid membranes and potentially affect membrane-associated signaling functions (**Figure 2**; Yu et al., 1992; Choi and Yu, 1995; Choe et al., 1995; Patel et al., 2001; reviewed in Piomelli et al., 2007). In addition, PLA_2_ mediated FFA liberation is the starting point for the generation of an extensive array of lipid-based neuromodulators. That includes FFAs themselves (e.g., AA and DHA) and LPLs (e.g., lysolecithin) but also encompasses an extensive array of biologically active molecules such as eicosanoids, isoprostanes, and neuroprostanes generated by a variety of lipid metabolizing pathways. Many of these pathways act as direct effectors of signal transduction in the nervous system through either direct interaction with ion channels or binding to G-protein-coupled or nuclear receptors (reviewed in Piomelli et al., 2007; Sultana et al., 2013).

Poly-unsaturated fatty acid peroxidation generally also leads to the formation of electrophilic aldehydes such as acrolein, malondialdehyde (MDA), 4-hydroxy-2-nonenal (4-HNE; Pamplona, 2008, 2011). These highly reactive aldehydes can seriously compromise molecular integrity of cells and tissues through the formation of molecular adducts with phospholipids, proteins, and DNA. However, recent evidence suggests that these lipid-derived aldehydes may also induce adaptive responses driven to decrease oxidative damage and improve antioxidant defenses (see for instance Pizzimenti et al., 2013).

## ANTIOXIDANT DEFENSES

Organisms are not defenseless against the actions of pro-oxidants. Rather, they posses a wide and varied array of enzymatic and non-enzymatic instruments to defend their structures and functions against oxidation that includes catalases, peroxidases, and dismutases, the GSH system and various other non-enzymatic anti-oxidants like vitamin C and vitamin E.

### ENZYMATIC MECHANISMS

Because of its relatively low redox potential and high abundance, the tripeptide GSH is a versatile anti-oxidant involved as primary redox buffering of many intra- and extra-cellular compartments, including lipid membranes, and mitochondria (Mari et al., 2009; Ribas et al., 2014). In addition to its antioxidant actions, GSH also plays an important role in detoxification processes, including the detoxification of potentially harmful products of lipid peroxidation such as aforementioned aldehydes. This process is mediated by members of the glutathione-*S*-transferase (GST) family of enzymes (Zimniak, 2008; Ribas et al., 2014). GSH is one of the most abundant molecules in healthy cells and tissues. Its availability is a function of usage, *de novo* synthesis and redox regeneration. The rate-determining step in GSH synthesis is catalyzed by the enzyme γ-glutamylcysteine synthetase (GCL; Chen et al., 2005; Genovese et al., 2007). Perturbation of GCL activity has been shown to significantly reduce GSH availability. In the context of the current focus of lipid bilayer redox homeostasis as a instrument of neuronal aging it is thought provoking that several studies report age-related declines in in GSH levels and GCL activity in various cell types, including neurons (Toroser and Sohal, 2005, 2007; Zhu et al., 2006; Jacob et al., 2013; Watson et al., 2014). This suggests that anti-oxidant failure either with or without escalation of pro-oxidant activity may be a key facet of the progressive lipid peroxidation stress characteristic of aging.

### NON-ENZYMATIC MECHANISMS

Non-enzymatic antioxidants such as Vitamins A, C, and E also play key roles in the defense against oxidative stress, including the maintenance of membrane redox homeostasis. Of these three vitamin families, Vitamin E is considered the major antioxidant present in membranes. Vitamin E is comprised of two families of closely related lipid-soluble compounds, the tocopherols, and tocotrienols. Alpha-tocopherol is the most abundant Vitamin E analog and according to recent insights has a preference for non-lipid raft PUFA-containing domains (Atkinson et al., 2010; Lemaire-Ewing et al., 2010). Alpha-tocopherol associates with membranes such that its active hydroxyl moiety is located at the lipid/water interface and not within the hydrocarbon matrix (Marquardt et al., 2013). As a consequence, α-tocopherol appears to exert its anti-oxidant activity at the surface of a membrane, possibly through the interception of diffusing ROS, or by means of terminating lipid radicals (Marquardt et al., 2013).

### NON-TRADITIONAL, NON-ANTIOXIDANT ACTIONS OF α-TOCOPHEROLS

In addition, several studies provide an alternative, non-antioxidant, scenario for α-tocopherol’s beneficial effects. That is, it is suggested that incorporation of α-tocopherols in membranes can structurally stabilize the membrane against lipid-peroxidation induced curvature stresses or normalize fatty acid signaling by acting as FFA trap through its ability to form hydrogen-bridges and engage in Vanderwaals interactions with FFAs (Atkinson et al., 2008; Lemaire-Ewing et al., 2010; Marquardt et al., 2013). Especially α-tocopherol’s acyl tail has been shown to play a critical role in its membrane stabilizing effect (Kagan, 1989). Methylation of this hydroxyl group disrupts α-tocopherol’s ability to engage in hydrogen-bridge formations. In this respect it is interesting to note that work from our lab shows that only non-methylated α-tocopherol is capable of reversing a decline in neuronal excitability due to age- or experimentally induced oxidative stress (Watson et al., 2012b).

## LIPID PEROXIDATION AND (BRAIN) AGING

Considering that neuronal membranes tend to have a high PUFA content it is not surprising that lipid peroxidation and its byproducts are increasingly implicated in brain aging. For instance, accumulation of MDA, 4-HNE, acrolein is commonly reported in aged brains of vertebrate and invertebrate model systems (Terman and Brunk, 2006; Zhu et al., 2006; Watson et al., 2012b). Moreover, PLA_2_s are also increasingly implicated in normal brain aging and the etiology of age-associated neurodegenerative disorders (Farooqui and Horrocks, 2006; Ong et al., 2010). How the products of PLA_2_ activity exert their detrimental effects in the aging brain is, however, not always clear but may involve one or more of the scenarios outlined in the preceding sections.

## INTRINSIC ELECTRICAL PROPERTIES AS A SUBSTRATE OF PLASTICITY

This review focuses on the role of intrinsic excitability of neurons in age-associated decline in behavioral plasticity. Decades of research on the cellular and molecular foundations of learning and memory have led to a “synapse-centric” view of learning and memory mechanisms that holds that memories are formed and stored through activity-dependent alterations of synaptic strength and neuronal circuit architecture (Bliss and Collingridge, 1993; Kandel, 2001). In this context non-synaptic mechanisms of plasticity, i.e., mechanisms involving changes in intrinsic electrical properties of neurons, have received much less attention. Yet, experimental evidence that changes in intrinsic neuronal electrical properties could be serving as part or whole of an engram beyond merely facilitating activity-dependent synaptic modulation goes back as far as the 1970s and the concept has gained substantial traction in recent years (reviewed in Benjamin et al., 2008; Mozzachiodi and Byrne, 2010; Sehgal et al., 2013; D’Angelo, 2014; for review of early work in this area see Byrne, 1987). For example, numerous studies have shown that eye blink trace conditioning in rodents induces an increase in intrinsic excitability of hippocampal CA1 and CA3 neurons through reduction of spike frequency adaptation caused by action potential afterhyperpolarizations (AHP) mediated by Ca^2+^-dependent outward K^+^ currents^2^ (Coulter et al., 1989; Moyer et al., 1992, 2000; Disterhoft et al., 1996; Thompson et al., 1996; Oh et al., 2010; Sehgal et al., 2014; for review, see Christian and Thompson, 2003). There is also substantial evidence of non-synaptic forms of plasticity in invertebrates, particularly in gastropod mollusks. For instance, Alkon (1974, 1979) demonstrated that associative learning in the nudibranch snail *Hermissenda crasicornis* involved an increase in excitability of type B photo-receptor cell associated with a decrease in the types of voltage- and Ca^2+^-sensitive K^+^ currents mediating the AHP and spike frequency adaptation. In *Lymnaea,* long-term memory (LTM) for classical conditioning of feeding has been associated with a persistent Na^+^-current dependent depolarization of the CGCs, two interneurons that act as gate-keepers to the feeding circuit and that play an essential role in chemosensory behavioral conditioning of the animals feeding behavior (Jones et al., 2003; Kemenes et al., 2006b; Nikitin et al., 2008, 2013). In the marine gastropod *Aplysia californica*, changes in intrinsic excitability of identified neurons in the central pattern-generating (CPG) network for feeding have been associated with the network’s behavioral conditioning induced motor output (Sieling et al., 2014).

More examples of non-synaptic plasticity can be found in the literature (see reviews by Byrne, 1987; Benjamin et al., 2008; Mozzachiodi and Byrne, 2010; Sehgal et al., 2013; D’Angelo, 2014). For the purpose of the current review it is suffice to say that the idea of intrinsic excitability regulation as a non-synaptic instrument of plasticity is now on firmer ground than ever before. The balance of this paper focuses on the idea that the mechanisms controlling intrinsic neuronal excitability, particularly those underlying the control of repetitive action potential firing, are a key target of oxidative stress-associated aging processes and a root cause of functional decline of normal aging neurons and brains.

## EVIDENCE FOR AGE-ASSOCIATED ALTERATIONS IN INTRINSIC ELECTRICAL PROPERTIES

Considering that neuronal electrical excitability is fundamental to brain function, substantial research on the neurobiological basis of behavioral aging has focused on the possibility that aging neurons undergo changes in intrinsic excitability. Neuronal excitability is a complex function of a neuron’s shape, the repertoire, distribution, and density of voltage- and ligand-gated ion channels in their plasma-membrane, the activity of ion pumps and various intra- and extracellular signaling systems and state variables, including intracellular Ca^2+^-, metabolic-, and redox homeostasis (Bean, 2007; Beck and Yaari, 2008; Vacher et al., 2008; Nusser, 2012). There is evidence for age-associated changes in many of these parameters. It is beyond the scope of this paper to review this vast and diverse literature. For a more in-depth treatment of this subject the reader is referred to many excellent reviews available in this area (see Barnes, 1994; Burke and Barnes, 2006; Mattson and Magnus, 2006; Chawla and Barnes, 2007; Thibault et al., 2007; Mattson et al., 2008; Oh et al., 2010; Toescu and Vreugdenhil, 2010; Yeoman et al., 2012). Instead in the next paragraphs we briefly review key insights gained from vertebrate and invertebrate model systems suggesting that intensification of spike frequency adaptation mechanisms is an important facet of the normal neuronal aging process.

### EVIDENCE FROM VERTEBRATE MODEL SYSTEMS

Most of the work on age-related intrinsic neuronal excitability changes in vertebrates has been done on the rodent hippocampus. On balance, the evidence from a large body of *in vitro* electrophysiological studies on different hippocampal regions of young and old mice, rats and rabbits suggest that most intrinsic neuronal electrical properties such as resting membrane potential, membrane time constant, input resistance, rheobase current, action potential threshold, and various aspects of action potential dynamics change very little across the lifespan (reviewed in Barnes, 1994; Burke and Barnes, 2006; Chawla and Barnes, 2007). However, “*a number of consistent changes do occur*” (Barnes, 1994). One of those changes involves alterations in voltage-gated Ca^2+^ currents, intracellular Ca^2+^-homeostasis, Ca^2+^-activated outward K^+^ currents and interactions between them enhancing the AHP and associated spike frequency adaptation of older neurons (Moyer et al., 1992; Tombaugh et al., 2005; reviewed in Oh et al., 2010). It should be noted, however, that even though the *in vitro* evidence for a role of these mechanisms in cognitive decline associated with normal brain aging is compelling, the idea is not undisputed (for review of this issue, see Burke and Barnes, 2006). Other studies report changes in voltage-activated Ca^2+^- or Na^+^-channel properties to age-associated decline in intrinsic excitability of various types of rodent neurons (Scott et al., 1988; Randall et al., 2012; Branch et al., 2014; for review see Annunziato et al., 2002; Foster, 2012).

### EVIDENCE FROM INVERTEBRATE MODEL SYSTEMS

Several studies, particularly in gastropod mollusks, have looked into the question of intrinsic neuronal excitability changes in invertebrate neurons. For example, Frolkis et al. (1991, 1995) reported increased voltage-gated Ca^2+^ current densities and changes in other aspects of intrinsic electrical excitability in aged *Lymnaea* neurons. The same group reported changes in voltage-gated K^+^ currents amplitudes and inactivation kinetics in old *Lymnaea* neurons (Frolkis et al., 1989). Patel et al. (2006) reported a decline in intrinsic excitability associated with a decline in input resistance and an increase in AHP magnitude and duration in CGGs of older *Lymnaea* and Klaassen et al. (1998) observed a decline in excitability associated with a reduction in input resistance in aging RPeD1. We observed a decline in intrinsic excitability of CGCs in aged learning-impaired older animals and concluded that “*this decline in excitability is not due to changes in passive neurophysiological properties but rather involves alterations in active properties of the cells”* (Hermann et al., 2007). An age-associated decline in input resistance associated with lengthening membrane time constant was reported for at least three identified neurons in *Aplysia* (Rattan and Peretz, 1981).

**FIGURE 7:**
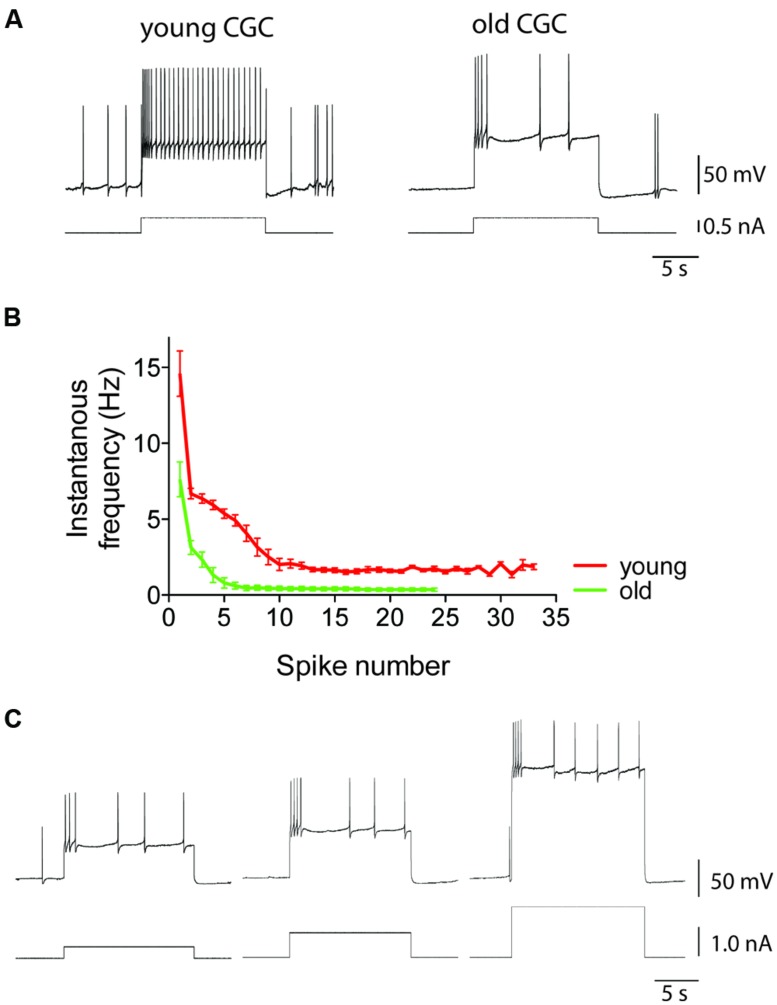
**Intrinsic electrical excitability of *Lymnaea* neurons declines with age.** Using CGCs as example, this figure illustrates the characteristic age-associated enhancement of spike frequency adaptation that is also observed in other identified *Lymnaea* neurons, including RPeD1 (see for instance Watson et al., 2012b, 2013). **(A)** This figure shows examples of intracellular recordings of spiking activity evoked in young and old CGCs by injection of 0.5 nA depolarizing current. Identical current injection results in a non-accommodating response in young CGCs but a rapidly accommodating response in old CGCs. **(B)** Average instantaneous frequency response of young and old CGCs. Current injected in young CGCs evokes a high-frequency action potential burst followed by a steady non accommodating firing response. In contrast, current injected in old CGCs evokes a few action potentials and a rapidly accommodating response. **(C)** Example of reduced excitability in old CGCs. Increasing stimulus strength fails to evoke a high-frequency non-accommodating response in old CGCs. Adapted from Hermann et al. (2007) with permission from American Psychological Association (APA).

Age-associated neurophysiological changes linked to behavioral impairment have been demonstrated in *Caenorhabditis elegans*, *Aplysia*, and *Lymnaea*. For instance, using a classical reward conditioning learning paradigm, we demonstrated in *Lymnaea* that a selective age-related impairment of appetitive LTM is associated with a decline in intrinsic excitability of the CGCs that is reflected in more pronounced spike frequency adaptation (**Figure 7**; Hermann et al., 2007). Along a similar vein, we also showed that age-related LTM impairment in aerial respiration operant-conditioning model is associated with enhanced spike frequency adaptation properties of RPeD1, an interneuron instrumental in the execution of this behavior and one of the cellular substrates of LTM in this learning paradigm (Watson et al., 2013). In addition, Janse et al. (1999) showed a correlation between reproductive senescence and reduced electrical excitability of the caudodorsal cells (CDCs), neuroendocrine cells producing *Lymnaea’s* ovulation hormone and other neuropeptides involved in the regulation of reproductive behavior (Janse et al., 1999). In *Aplysia*, studies from the Peretz lab and a recent study by Kempsell and Fieber (2014) have linked age-associated behavioral and homeostatic deficiencies to declining neuronal excitability (Rattan and Peretz, 1981; Peretz, 1988; Skinner and Peretz, 1989). Interestingly, in the nematode *C. elegans* age-associated decline in chemotactic behavior is associated with a decline in sensory neuron excitability due to oxidation-induced enhancement of voltage-gated K^+^ currents (Cai and Sesti, 2009; Sesti et al., 2010).

## HOW DOES OXIDATIVE STRESS CONTRIBUTE TO DECLINING EXCITABILITY OF AGING NEURONS?

How oxidative stress translates into functional decline and increased vulnerability to disease of the aging brain is only partly understood (Mattson and Magnus, 2006). First it should be noted that in the context of redox modulation of neuronal functions many pro-oxidants have both “good” and a “bad” aspects. For instance, under normal conditions pro-oxidants like RONS play important physiological roles in synaptic plasticity and memory formation (reviewed in Massaad and Klann, 2011; Milton and Sweeney, 2011). Yet, under conditions of oxidative stress the same mechanisms may affect neuronal plasticity and memory function in a negative manner (Hu et al., 2006; Negre-Salvayre et al., 2010; Massaad and Klann, 2011; Niki, 2012).

Second, many of the molecular instruments of neuronal excitability (i.e., membrane ion channels, receptors, vesicle fusion processes) are susceptible to oxidation or sensitive to alterations in cellular redox status (Annunziato et al., 2002; Dukoff et al., 2014). For instance, a particularly salient example concerns the so-called A-current (I_A_) family, a family of outward transient voltage-gated K^+^ currents (i.e., they are self-inactivating) that plays important roles in the regulation of neuronal excitability (Adams and Galvan, 1986; Bean, 2007). It has long been known that I_A_’s inactivation properties are modulated by changes in intracellular redox conditions (Ruppersberg et al., 1991; Bähring et al., 2001). That is, under cytosolic reducing conditions the current displays its characteristic fast N-type inactivation behavior whereas under oxidizing conditions this inactivation process is slowed down. In a manner of speaking, this process turns I_A_ from a “temporary brake” into a “permanent brake” on a neuron’s spiking ability. It has been suggested that these mechanisms may operate as a safety switch against metabolic overload by matching a neuron’s energy demanding electrical activity to its metabolic capacity (Gulbis, 2002). The I_A_ family of ion channels is not the only type of ion channels susceptible to changes in cellular redox homeostasis. Many other types of ion channels playing significant roles in the control and execution of neuronal excitability, including voltage-gated Na^+^ and Ca^2+^, are subject to redox modulation (for review see Annunziato et al., 2002).

In addition, over the last number of decades it has become clear that many of the molecular correlates of neuronal excitability and processes are sensitive to the composition and micro-architecture of their membrane environment and/or are modulated by various membrane-derived lipid signaling moieties (for review see Piomelli et al., 2007; Dart, 2010; Rosenhouse-Dantsker et al., 2012). Maintaining lipid membrane homeostasis is therefore a crucial facet of the maintenance of functional integrity of neurons and the brain. Below we illustrate these ideas with some of the most salient examples.

## MEMBRANE-ASSOCIATED CORRELATES OF NEURONAL EXCITABILITY CONTROL

There are numerous examples of lipid-mediated signaling in the nervous system. For instance, although there is still some debate whether its actions arise from direct or indirect interactions with ion channel proteins it is evident that AA, one of the most common PUFAs in the nervous system, can modulate a variety of ion channels, including voltage-gated Ca^2+^ channels, mechano-gated K^+^ channels like TRAAK, TREK-1, and other members of the four-transmembrane/two-pore domain (4TM2P) family of background K^+^ channels (Patel et al., 1998; Maingret et al., 1999; Roberts-Crowley et al., 2009). Alternatively, PUFAs like AA or EPA liberated from their plasma membrane environment can act as metabolic precursors for several signaling pathways active in the nervous system. This includes the eicosanoid pathway that generates a collection of signaling molecules (i.e., prostaglandins, prostacyclins, thromboxanes, lipoxins, and leukotrienes). Many eicosanoids regulate immune functions and inflammation but also double as neuromodulators (for review see Nigam and Schewe, 2000; Liang et al., 2007). Intriguingly, one of those eicosanoid neuromodulators, the 12-lipoxygenase (12-LOX) metabolite 12-HPETE has long been known as an activator of *Aplysia*’*s* S-type K^+^-channels (Piomelli et al., 1987). These K^+^ channels, later identified to belong to the TREK-1 family, are instrumental in non-synaptic forms of plasticity underlying behavioral modification of *Aplysia*’s gill withdrawal reflex (Hawkins, 1984) Similar PLA_2_- and/or LOX-dependent arachidonic-acid modulated background K^+^ channels have been described in *Lymnaea* (Kits et al., 1997; Lopes et al., 1998). Opening of these 4TM2P channels generates an outward current. It is, therefore, conceivable that elevated levels of PLA_2_-activity associated with inflammation, oxidative stress, and aging induce a decline in neuronal excitability through activation of these 4TM2P background K^+^ channels. An alternative route linking membrane oxidative stress to a decline in neuronal excitability involves aforementioned aldehyde byproducts of lipid peroxidation. As mentioned MDA, 4-HNE and acrolein can cause irreversible damage to many of the cells macromolecules through covalent conjugation (Farooqui and Horrocks, 2006) or engage in various signaling activities including pathways regulating mitochondrial function and other aspects of cell metabolism (Riahi et al., 2010; Ayala et al., 2014).

Yet another intriguing plausible explanation for the effects of lipid peroxidation on neuronal excitability involves structural rather than signaling arguments. Specifically, as noted before peroxidation very likely incites considerable changes in the molecular geometry of affected PUFAs that affect the way they “fit” in the membrane and may introduce considerable changes in the micro-architecture and biophysical properties of bilayer membranes including parameters such as membrane curvature, membrane viscosity, lipid composition, and distribution. These changes may have various implications for neuronal signaling functions, including the organization of transmembrane signaling complexes, vesicle fusion processes, receptor trafficking, and ion channel functions (Yu et al., 1992; Choi and Yu, 1995; Choe et al., 1995; Patel et al., 2001; reviewed in Piomelli et al., 2007). For example, membrane curvature stresses have been found an important factor in the regulation of neuronal electrical activity through the activation of stretch/mechano sensitive background K^+^ channels known to contribute to the regulation of neuronal excitability (**Figure 2**; Patel et al., 2001). Also, the lipid architecture of presynaptic terminal membranes does play a vital role in synaptic Ca^2+^-triggered exocytosis process raising the possibility that PUFA peroxidation interferes with synaptic functions (reviewed in Davletov and Montecucco, 2010).

Taken together, even though there are still a lot of loose ends in the conceptual framework we sketched here it seems evident that proper management of membrane redox status, lipid peroxidation and phospholipid lysis products generated by or with the aid of PLA_2_ is likely critical for functional homeostasis of neurons and by extension the nervous system. Below we summarize and discuss evidence from recent work done in our laboratory trying to fit the pieces of this puzzle together.

## PLA_2_ – A LIPASE AT THE NEXUS OF AGING, OXIDATIVE STRESS, NEURONAL ELECTRICAL EXCITABILITY CONTROL AND FUNCTIONAL DECLINE OF THE AGING NERVOUS SYSTEM?

In the preceding paragraphs we explored the prospect for interplay between oxidative stress, lipid membrane factors, PLA_2_, and changes in intrinsic neuronal excitability as a factor in functional decline of normal aging neurons. In this section we review the evidence that lipid peroxidation-induced PLA_2_ activity lays at the root of inflammation-, age-, and oxidative stress-associated functional impairment of the nervous system in *Lymnaea* and put our findings in the context of a growing literature suggesting that we are dealing with an evolutionary widely conserved mechanism.

**FIGURE 8:**
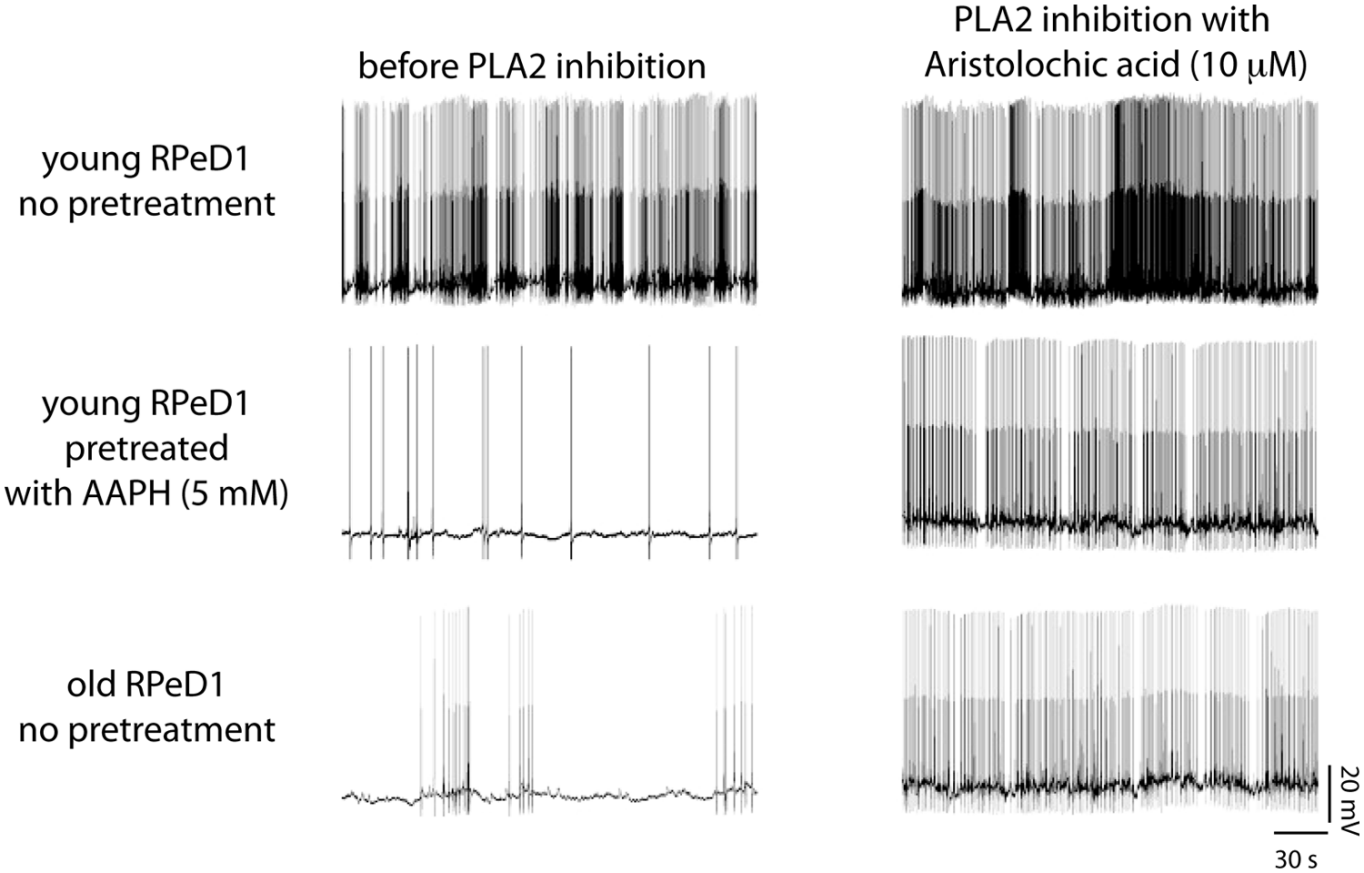
**Phospholipase A_2_ (PLA_2_) contributes to regulation of respiratory neural circuit activity in old and oxidative stressed young brains.** Examples of action potential activity recorded from RPeD1 in isolated young central nervous systems (CNSs) pretreated for 30 min with either vehicle-only **(top row)** or the pro-oxidant 2,2-azobis (2-methylpropion-amidine) dihydrochloride (AAPH; **middle row**), and old CNSs maintained in vehicle-only **(bottom row)**, before **(left column)**, and after 30 min of treatment with the PLA_2_ inhibitor aristolochic acid **(right column)**. Reprinted from Watson et al. (2013) with permission from Elsevier.

As described above, aging *Lymnaea* display associative LTM impairment in two functionally and anatomically distinct (i.e., appetitive and respiratory) learning paradigms (Hermann et al., 2007, 2013; Watson et al., 2012a, 2013, 2014; Beaulieu et al., 2014). In both instances, LTM failure was associated with reduced spontaneous action potential activity and enhanced spike frequency adaptation of the two key interneurons underlying both execution and plasticity of the respective behaviors (i.e., CGCs and RPeD1). Both behavioral and electrophysiological facets of age-associated respiratory and appetitive LTM impairment could be reproduced in young animals through treatment with 2,2′- azobis-2-methyl-propanimidamide (AAPH), a water-soluble free radical generator commonly used to induce PUFA peroxidation, furthering the notion of plasmamembrane lipid peroxidation as a significant agent of neuronal and behavioral aging in *Lymnaea* (**Figure 8**). Evidence of increased FFA mobilization from brains and other tissues was found in both old and AAPH-treated young animals (Watson et al., 2013; Beaulieu et al., 2014). Moreover, treatment with the lipid-domain anti-oxidant α-tocopherol reversed decline in neuronal excitability in both experimental oxidative stressed and naturally aged neurons (Watson et al., 2012a,b). Remarkably, all these behavioral, electrophysiological, and biochemical symptoms of aging and experimental oxidative stress could be reversed by treatment with aristolochic acid, a broad spectrum PLA_2_ inhibitor that inhibits both Ca^2+^-dependent cPLA2 and Ca^2+^-independent iPLA_2_ (**Figure 8**; Vishwanath et al., 1988; Lopes et al., 1998; Carnevale and Cathcart, 2001).

No evidence was found of significant age-associated impairment or experimental oxidative stress-induced repression of transcription-independent short/intermediate term memory (S/ITM) in either of the two behavioral conditioning paradigms, suggesting that transcription-independent forms of memory appear impervious to aging or oxidative stress. As yet, we have no definitive answer to the question why LTM is selectively susceptible. However, preliminary evidence from our laboratory suggests that both aging and AAPH-induced oxidative stress in participation with PLA_2_ alters aspects of mitochondrial functions that may affect their ability to contribute to intracellular Ca^2+^-buffering. The molecular pathways underlying LTM formation in *Lymnaea* involve “traditional” conserved cAMP- and Ca^2+^-sensitive CREB- and MAPK-dependent pathways (Ribeiro et al., 2003; Wagatsuma et al., 2006; Guo et al., 2010). It is therefore conceivable that dysregulation of intracellular Ca^2+^ homeostasis disrupts transcription-regulation pathways involved in LTM expression. Furthermore, the observation that PLA_2_ inhibition rescues LTM is particularly intriguing in view of evidence implicating FFAs and PLA_2_ in mitochondrial uncoupling in pancreatic β-cells (Ježek et al., 2010, 2014). Although speculative at this point in time, it is not unreasonable to postulate that a similar mechanism may underlie the PLA_2_-associated LTM impairment in *Lymnaea*.

Intriguingly, selective appetitive LTM impairment could also be induced in young *Lymnaea* through systemic activation of their cellular immune system (Hermann et al., 2013). As before in naturally aged and experimentally oxidation-stressed young animals, inflammation-associated LTM impairment could be rescued by means of PLA_2_ inhibition (Hermann et al., 2013). Together, our data indicate that the processes underlying *Lymnaea’s* transcription-dependent forms of memory are particularly vulnerable to aging, oxidative stress, and inflammation and that PLA_2_ plays a pivotal role in each of these models of associative LTM failure.

## PLA_2_’s AS THERAPEUTIC TARGETS?

PLA_2_’s and deregulation of lipid metabolism are increasingly implicated in human age-, oxidative stress-, and inflammation-associated conditions including cardiovascular diseases and age-associated neurodegenerative diseases such as Alzheimer’s disease (Sun et al., 2004; Farooqui and Horrocks, 2006; Adibhatla and Hatcher, 2008; Sanchez-Mejia et al., 2008; Chalimoniuk et al., 2009; Sanchez-Mejia and Mucke, 2010; Desbene et al., 2012; Gentile et al., 2012; Hui, 2012; reviewed in Sun et al., 2014). They are therefore, not surprisingly, increasingly targeted or considered as targets for therapeutic intervention (see for instance, Garcia-Garcia and Serruys, 2009; Quach et al., 2014). Particularly, it is intriguing that lipoprotein-associated phospholipase A_2_ (Lp-PLA_2_), also known as platelet-activating factor acetylhydrolase (PAF-AH) was identified as a potential factor in cerebrovascular oxidative stress and inflammation and risk factor for human dementia and subsequently drew considerable interest as a therapeutic target in Alzheimer’s disease (Davidson et al., 2012; Acharya et al., 2013; Fitzpatrick et al., 2014). However, there are still a lot of unknowns surrounding the pathways, processes and conditions leading to PLA_2_ activation in different cell types that should be resolved before effective and safe therapeutic targets and agents for the treatment and prevention of these increasingly germane age-associated public health concerns (Sun et al., 2014). For instance, whereas many studies implicate PLA_2_ hyperactivity as a factor in the etiology of Alzheimer’s disease (Sanchez-Mejia and Mucke, 2010; Gentile et al., 2012; Hui, 2012), other studies suggest that hypoactivity of intracellular PLA_2_s (cPLA_2_ and iPLA_2_) are a contributing factor to memory impairment and Alzheimer’s neuropathology (reviewed by Schaeffer et al., 2009).

## RELEVANCE AND FUTURE DIRECTIONS

The evidence reviewed here infers close ties between inflammation, oxidative stress, lipid-peroxidation, PLA_2_-activation, neuronal excitability, and AMI in *Lymnaea* and echo a rapidly growing evidence from basic, clinical, and epidemiological research that PLA2s, inflammation and oxidative stress are important factors in age-related cognitive decline and impairment in humans and other mammals (Sun et al., 2004, 2014; Phillis et al., 2006; Adibhatla and Hatcher, 2008; Lee et al., 2011). Considering this remarkable conservation we believe that *Lymnaea*’s extraordinary suitability for detailed, direct, and tightly integrated molecule-to-behavior dissection of the neurobiological substrates of AMI will in addition to furthering understanding of the biological foundations and parameters of aging potentially also generate valuable insights into mechanisms of and possible solutions for human AMI and other age-associated aﬄictions of our own nervous system. Before that goal can be achieved many questions need to be answered.

Among the unanswered questions requiring further attention is the identity of the ionic mechanisms through which the products of PLA_2_-mediated fatty acyl hydrolysis exert their inhibitory influence on neuronal excitability. As outlined in the preceding pages, there are many possibilities to test ranging from FA dependent modulation of ion channels to alterations in autacoid modulation of synaptic functions and hypotheses encompassing elements of membrane architecture dependent regulation of neuronal electrical properties.

Another important puzzle to solve is what inhibition of PLA_2_, one of the main tools of membrane homeostasis and repair, does to long-term cell/tissues homeostatic integrity. Considering the impact lipid peroxidation is expected to have on membrane structure and function, our finding that PLA_2_ inhibition restored rather than aggravated neuro-physiological and behavioral correlates of aging, experimental oxidative stress, and inflammation is puzzling and urges further study.

Yet another intriguing perspective we are currently exploring is mitochondrial involvement in the lipid peroxidation- and PLA_2_-dependent declining excitability and plasticity of aging *Lymnaea* neurons. Neurons critically depend on mitochondria to provide the energy to establish and maintain membrane excitability and engage in their quintessential and metabolically costly electrochemical signaling activities (Kann and Kovács, 2007; Mattson et al., 2008). Mitochondria are also a critical partner in intracellular free Ca^2+^ homeostasis and are one of the main sources of RONS and other pro-oxidants. There are many tight reciprocal links between neuronal electrical and mitochondrial activities and many neuronal excitability disorders appear to be associated with mitochondrial aberrancies (Kann and Kovács, 2007; Mattson et al., 2008; Surmeier et al., 2012). Moreover, there is also a substantial body of evidence indicating involvement of mitochondria-associated oxidative stress, lipid signaling, Ca^2+^ dysregulation in normal aging, and the pathogenesis of common age-associated neurological diseases including AD and PD in which lipid peroxidation and PLA_2_ may be key partners (reviewed in Thibault et al., 2007; Mattson et al., 2008; Toescu and Vreugdenhil, 2010; Waldbaum and Patel, 2010). The *Lymnaea* model system we have developed provides an excellent platform to investigate these interactions between neuronal energy metabolism, electrical excitability, and plasticity in great detail.

The *Lymnaea* research platform we portrayed here will also be a great tool to investigate fundamental mechanisms of neuronal and behavioral aging and test other existing and emerging aging theories. For example, there is growing evidence that insulin and other insulin-like peptides (ILPs) modulate aspects of plasticity in the *Lymnaea* CNS and enhance learning abilities in older learning-impaired snails (Murakami et al., 2013; Pirger et al., 2014). These findings resonate very well with a rapidly expanding literature that ILPs and insulin resistance are a major endocrine factor in aging in general and aging of the nervous system in particular (Kim and Feldman, 2012; Alcedo et al., 2013). Moreover, because of its identified neurons the model system will allow us to explore many important fundamental scientific questions about aging neurons and brains like: do neurons age according to “plan” or do they fail at random? Are some neuronal physiological phenotypes more vulnerable to aging than others? Can we reverse the effects of aging without repercussion? What are the principles and mechanisms underlying robust and reliable complex brains? Are all changes observed in aging neurons mal-adaptive or are some perhaps compensatory and adaptive? Are neurons and brains life cycles subject to the same resource constraints and trade-offs as other types of cells and tissues in the animal body?

## Conflict of Interest Statement

The authors declare that the research was conducted in the absence of any commercial or financial relationships that could be construed as a potential conflict of interest.

## Footnotes

1 Key concept 1 | Methylene-interrupted PUFAs. Poly-unsaturated fatty acids with one or more pairs of hydrogen unsaturated carbons in *cis*-arrangement separated by a hydrogen-saturated (bis-allylic) methylene bridge. Because the methylene moiety’s bis-allylic C–H bonds are weaker than ordinary C–H bonds they are prone to free radical attack. The main PUFAs with more than one bis-allylic methylene site in the plasmamembranes of neurons are arachidonic acid (AA), docosahexanoic acid (DHA), and eicosapentaenoic acid (EPA).

2 Spike frequency adaptation, Action potential afterhyperpolarization (AHP), Ca^2+^-activated K^+^ currents – Spike frequency adaptation is a process by which the action potential firing rate of a neuron declines with time despite the maintenance of a constant excitatory stimulus. The action potential of most neurons is followed by an HP, a period of varying length of time during which the cells’ membrane potential is lower than the resting potential preceding the action potential and during which the cell is less excitable than before. Various ionic mechanisms may contribute to the AHP including a class of outward K^+^ currents activated either exclusively or in part by increase of intracellular free Ca^2+^ levels (i.e., Ca^2+^-activated K^+^ currents). The AHP as part of a negative feedback loop linking spiking frequency and opening of voltage-gated Ca^2+^ channels to activation of inhibitory Ca^2+^-activated K^+^ currents is a key component of spike frequency adaptation in most neurons (see Lewis et al., 1986; Sah, 1996; Sah and Davies, 2000 for more detail).
